# Broadening horizons: ferroptosis as a new target for traumatic brain injury

**DOI:** 10.1093/burnst/tkad051

**Published:** 2024-01-18

**Authors:** Ziqing Wei, Haihan Yu, Huijuan Zhao, Mingze Wei, Han Xing, Jinyan Pei, Yang Yang, Kaidi Ren

**Affiliations:** Department of Neurology, The First Affiliated Hospital of Zhengzhou University, No. 1, Jianshe East Road, Erqi District, Zhengzhou, China; Henan Key Laboratory of Cerebrovascular Diseases, The First Affiliated Hospital of Zhengzhou University, No. 1, Jianshe East Road, Erqi District, Zhengzhou, China; Clinical Systems Biology Laboratories, The First Affiliated Hospital of Zhengzhou University, No. 1, Longhu Middle Ring Road, Jinshui District, Zhengzhou, China; Department of Neurology, The First Affiliated Hospital of Zhengzhou University, No. 1, Jianshe East Road, Erqi District, Zhengzhou, China; Henan International Joint Laboratory of Thrombosis and Hemostasis, College of Basic Medicine and Forensic Medicine, Henan University of Science and Technology, No. 1, Longhu Middle Ring Road, Jinshui District, Luoyang, China; The Second Clinical Medical College, Harbin Medical University, No. 263, Kaiyuan Avenue, Luolong District, Harbin, China; Department of Pharmacy, the First Affiliated Hospital of Zhengzhou University, No. 246, Xuefu Road, Nangang District, Zhengzhou 450052, China; Henan Key Laboratory of Precision Clinical Pharmacy, Zhengzhou University, No. 1, Jianshe East Road, Erqi District, Zhengzhou 450052, China; Quality Management Department, Henan No.3 Provincial People’s Hospital, No. 198, Funiu Road, Zhongyuan District, Henan province, Zhengzhou 450052, China; Clinical Systems Biology Research Laboratories, Translational Medicine Center, the First Affiliated Hospital of Zhengzhou University, No. 198, Funiu Road, Zhongyuan District, Zhengzhou 450052, China; Department of Pharmacy, the First Affiliated Hospital of Zhengzhou University, No. 246, Xuefu Road, Nangang District, Zhengzhou 450052, China; Henan Key Laboratory of Precision Clinical Pharmacy, Zhengzhou University, No. 1, Jianshe East Road, Erqi District, Zhengzhou 450052, China

**Keywords:** Traumatic brain injury, Ferroptosis, Therapeutic targets, Lipid peroxidation, Reactive oxygen species, Metabolism, Blood-brain barrier

## Abstract

Traumatic brain injury (TBI) is a leading cause of death and disability worldwide, with ~50 million people experiencing TBI each year. Ferroptosis, a form of regulated cell death triggered by iron ion-catalyzed and reactive oxygen species-induced lipid peroxidation, has been identified as a potential contributor to traumatic central nervous system conditions, suggesting its involvement in the pathogenesis of TBI. Alterations in iron metabolism play a crucial role in secondary injury following TBI. This study aimed to explore the role of ferroptosis in TBI, focusing on iron metabolism disorders, lipid metabolism disorders and the regulatory axis of system Xc^−^/glutathione/glutathione peroxidase 4 in TBI. Additionally, we examined the involvement of ferroptosis in the chronic TBI stage. Based on these findings, we discuss potential therapeutic interventions targeting ferroptosis after TBI. In conclusion, this review provides novel insights into the pathology of TBI and proposes potential therapeutic targets.

Highlights

- We concluded the role of ferroptosis in TBI, focusing on iron metabolism and lipid metabolism disorder, and the regulatory axis of System Xc-/GSH/GPX4 in TBI.
- We indicated the involvement of ferroptosis in the chronic TBI stage.
- We discussed the potential therapeutic interventions targeting ferroptosis after TBI.

## Background

**Figure 1:**
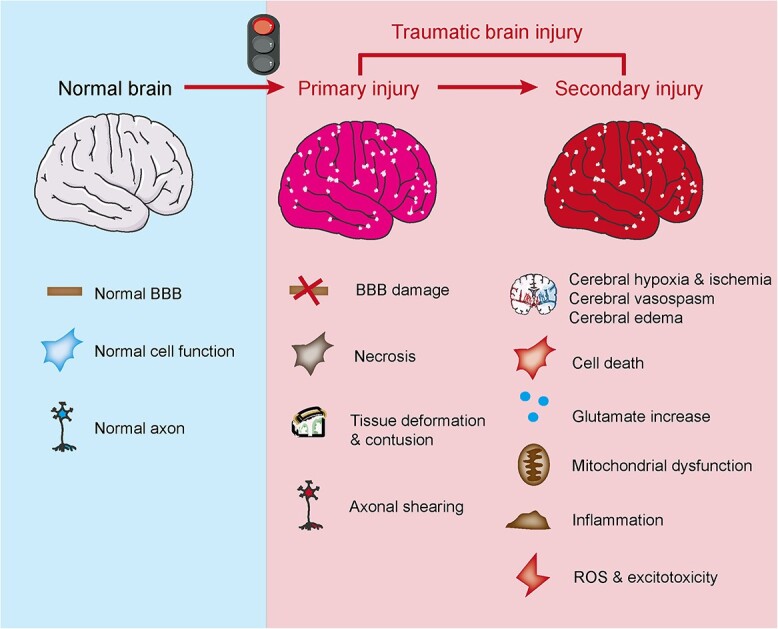
Pathophysiology of TBI. TBI can be classified into two distinct injury phases based on pathophysiology: primary injury and secondary injury. Primary injury refers to immediate damage to brain tissue, including tissue deformation, contusion, necrosis and axonal shearing. Mechanical force disrupts the integrity of the BBB. Subsequently, the initial mechanical trauma can initiate a cascade of events leading to secondary injury, which encompasses increased intracranial pressure, mitochondrial dysfunction, inflammation, reactive oxygen species (ROS), excitotoxicity, cell death, ischemia and other processes. *TBI* traumatic brain injury, *BBB* blood–brain barrier

Traumatic brain injury (TBI) is a prominent global cause of death and disability, imposing a significant economic burden. Annually, ~50 million people worldwide experience TBI, with an estimated 50% of the population likely to suffer from it at least once in their lifetime [1]. The primary causes of TBI include traffic accidents, falls from heights, violent attacks, and slips and falls [2]. TBI can be classified into two categories based on the timing and nature of the injury: primary injury and secondary injury [3,4] (Figure 1). Primary injury results directly from external forces and encompasses the disruption of the blood–brain barrier (BBB), damage to axonal fibers and cellular death [5]. Secondary injury refers to a cascade of tissue damage triggered by biochemical changes following the primary injury. Its underlying mechanisms are complex, encompassing excitatory neurotoxicity, oxidative stress, intracellular calcium overload, inflammatory response, apoptosis and other processes [6]. While primary injury is irreversible, secondary injury can be mitigated [7]. Consequently, reducing neuronal death represents a pivotal strategy in treating secondary injury in TBI and serves as a pathophysiological mechanism and potential therapeutic target.

Ferroptosis, a recently identified form of programmed cell death, primarily occurs due to mitochondrial changes induced by excessive accumulation of intracellular lipid peroxides and iron dependence [8]. Research indicates a potential association between ferroptosis and traumatic central nervous system conditions, suggesting its involvement in the pathogenesis of TBI both *in vivo* and *in vitro* [9]. In animal models of TBI, iron overload, elevated transferrin expression, lipid reactive oxygen species (ROS) accumulation and mitochondrial atrophy associated with iron metabolic pathways have been observed [9,10]. Notably, treatment with the inhibitor ferrostatin-1 (Fer-1), which targets ferroptosis, has demonstrated a reduction in neuronal death and improvements in long-term cognitive and motor functions, further confirming the existence of ferroptosis [9].

In this review, we will focus on the mechanisms of iron and ferroptosis involved in TBI and elaborate on the potential value and research progress of targeted ferroptosis therapy for TBI. Through this review, we can better find new clinical treatment directions targeting iron and ferroptosis after TBI.

## Review

### The role of ferroptosis in TBI

**Figure 2:**
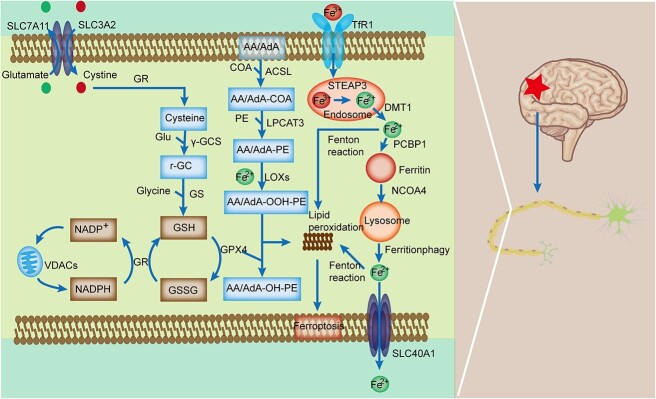
The mechanisms of ferroptosis in TBI. In the iron metabolism pathway, extracellular Fe^3+^ enters cells through endocytosis, combines with TF and TFR1 and is subsequently stored in the endosome. At low pH, Fe^3+^ is reduced to Fe^2+^ and transported to the cytoplasm by DMT1 with the cooperation of STEAP3. It is then stored in ferritin through PCBP1, and some is transported out of the cell via SLC40A1. Fe^2+^ is released through the ferritinophagy pathway, mediated by NCOA4. Following TBI, Fe^2+^ overload leads to the generation of a large amount of ROS through the Fenton reaction. In the lipid metabolism pathway, PUFAs such as AA and AdA in the cytomembrane become susceptible to oxidizing substances after TBI. PUFAs can transform into fatty acid hydroperoxides, ultimately leading to ferroptosis under the catalysis of ACSL4, LPCAT3, LOXs and other biological enzymes. System Xc^−^ is a cystine/glutamate antiporter that transports cystine into cells and glutamate out of cells. Intracellularly, cystine is reduced to cysteine for GSH biosynthesis. GSH is needed for GPX4 activity and reverses lipid peroxidation. Glutathione disulfide (GSSG) is recycled by GSH reductase in an NADPH-dependent manner, and the oxidation state of NADH is determined by the voltage-dependent anion channel (VDAC). TBI leads to GSH depletion and decreases GPX4 activity, increasing lipid peroxides. *TBI* traumatic brain injury, *TF* transferrin, *TFR1* TF-iron-TFR1 with transferrin receptor 1, *ROS* reactive oxygen species,* DMT1* divalent metal transporter 1, *AA *arachidonoyl, *AdA* adrenoyl, *PUFAs* Polyunsaturated fatty acids, *LOXs* lipoxygenases, *GSH* glutathione, *PEs *phosphatidylethanolamines

Ferroptosis is a type of regulated cell death induced by iron ion-catalyzed and reactive oxygen species-induced lipid peroxidation that was initially proposed by Professor Brent R. Stockwell of Columbia University in 2012 [8]. Unlike classical apoptosis, necrosis and autophagy, ferroptosis does not involve cell shrinkage or chromatin condensation but instead exhibits mitochondrial shrinkage, increased membrane density, decreased cristae and heightened lipid peroxidation [8]. Ferroptosis has been associated with pathological cell death in various degenerative diseases, such as Alzheimer's disease (AD), Huntington's disease (HD) and Parkinson's disease (PD), as well as cancer, stroke, cerebral hemorrhage, renal degeneration, spinal cord injury (SCI) and TBI [11–13]. Interestingly, ferroptosis also exhibits a potential tumor suppressor effect [14,15]. Growing evidence highlights the significant role of ferroptosis in the development and progression of numerous diseases, making it a promising target for various pharmacological treatments [11,16]. The regulatory mechanisms of ferroptosis are intricate and involve iron homeostasis, lipid peroxidation and imbalances in the amino acid antioxidant system [17] (Figure 2).

### Iron metabolism disorder

Iron is an essential trace element and the most abundant element in the body [18]. It plays a vital role in numerous physiological and biochemical functions and is a key contributor to ferroptosis [8]. Under normal conditions, extracellular Fe^3+^ ions initially bind to transferrin (TF), forming a complex known as TF-iron-TFR1 with transferrin receptor 1 (TFR1) [19]. This complex is subsequently internalized by clathrin-coated pits. Within acidic vesicles, Fe^3+^ ions undergo reduction to Fe^2+^ and are then transported to the labile iron pool (LIP) in the cytoplasm through the action of divalent metal transporter 1 (DMT1) [8,19]. Surplus iron is stored as a complex composed of ferritin light-chain subunits and ferritin heavy-chain subunits. Meanwhile, any remaining Fe^2+^ is oxidized to Fe^3+^ and exported from the cell to contribute to the body’s iron recycling process [20]. Damage or ischemia–hypoxia in tissues disrupts cellular metabolism, accumulating a substantial quantity of Fe^2+^ ions. Fe^2+^ ions engage in Fenton reactions with hydrogen peroxide, producing destructive hydroxyl radicals [21]. These radicals can interact with cellular lipid components, triggering lipid peroxidation and generating numerous lipid radicals [21]. This process intensifies the disruption of cell membrane structure, elevates cell membrane permeability, and ultimately culminates in cell rupture and death [22].

**Figure 3:**
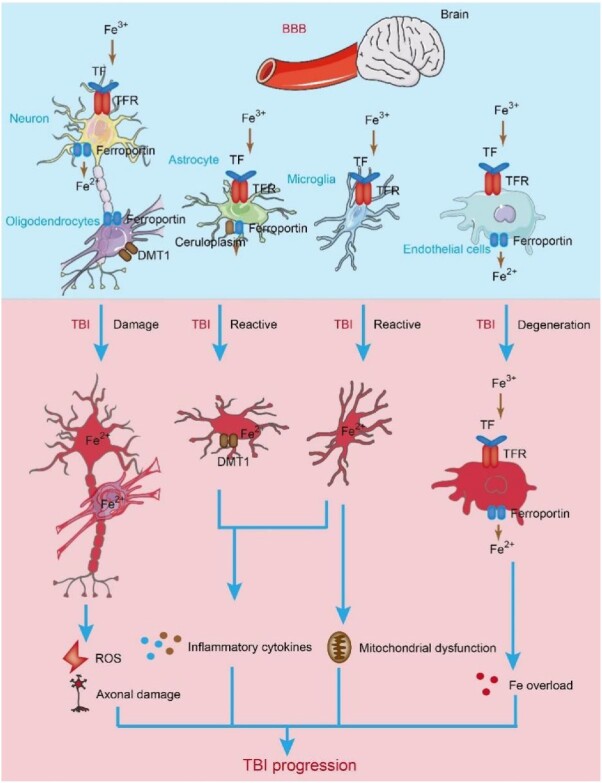
Iron metabolism in the brain after TBI. Iron needs to traverse the BBB to enter the brain. It is taken up from the blood by brain capillary endothelial cells and enters endosomes through internalization and binding with TF and TFR on endothelial cells. DMT1 binds to Fe^2+^ and transports it into labile LIP in the cytoplasm. In an acidic environment, Fe^2+^ dissociates from the Fe^3+^-TF-TFR complex, undergoes oxidation to Fe^3+^ and enters the cytoplasm through DMT1. It is then transported out of endothelial cells with membrane transport proteins and ferroxidase. Iron ions in the brain parenchyma are mainly taken up, utilized or stored by various neural cells. Astrocytes, which do not express TFR, primarily take up Fe^3+^ through a nontransferrin-dependent pathway. Once inside neural cells, Fe^2+^ can enter mitochondria and participate in the redox reactions of the mitochondrial respiratory chain. Excess Fe^2+^ can be oxidized to Fe^3+^ by the heavy chain of ferritin and stored in oligodendrocytes and astrocytes. After the occurrence of TBI, free iron levels increase, disrupting iron homeostasis and leading to iron overload in brain tissue. Reactive oxygen species are generated through the Fenton reaction, causing oxidative stress and damaging neural cells. Astrocytes and microglia release proinflammatory cytokines, which can induce oxidative damage or cell death in neurons and glial cells. *TBI* traumatic brain injury, *BBB* blood–brain barrier, *TF* transferrin, *TFR1* TF-iron-TFR1 with transferrin receptor 1, *ROS* reactive oxygen species, *DMT1* divalent metal transporter 1

Recent studies have shown that alterations in iron metabolism are important mechanisms underlying secondary injury following TBI [23,24] (Figure 3). For example, iron deposition and abnormal iron metabolism have been observed in a controlled cortical impact (CCI) mouse model, and intraventricular injection of Fer-1 could significantly reduce iron accumulation and neuronal damage, improving long-term outcomes [23].

### Lipid metabolism disorder

It is now understood that the disruption of iron-dependent lipid oxidation metabolism is a pivotal factor in ferroptosis [16]. Polyunsaturated fatty acids (PUFAs) constitute the predominant components of cellular and organelle membrane phospholipids and play a critical role as substrates for the synthesis of phosphatidylethanolamines (PEs) [25], which are indispensable for maintaining the fluidity and structural integrity of cell membranes [26]. Moreover, PUFAs significantly contribute to the accumulation of lipid peroxides during the process of ferroptosis [27]. Specifically, PUFAs such as arachidonoyl (AA) or adrenoyl (AdA) [26], exhibit the highest susceptibility to oxidation and are precisely regulated by three synthesizing enzymes [26]. acyl-CoA synthetase long-chain family 4 (ACSL4) catalyzes the conversion of AA or AdA into AA-CoA and AdA-CoA [28], which are subsequently esterified by lysophosphatidylcholine acyl-transferase 3 (LPCAT3) to form AA-PE and AdA-PE [29]. Finally, arachidonate lipoxygenases (ALOXs) or cytochrome P450 oxidoreductases oxidize AA-PE and AdA-PE, leading to the generation of lipid peroxides [30,31]. Several studies have demonstrated that knockout or inhibition of these three synthesizing enzymes can effectively suppress the occurrence and progression of ferroptosis [30–32]. These aldehydes induce protein carbonylation, leading to aberrant protein structure and impaired functionality [27]. Ultimately, this cascade of events culminates in irreversible damage to the structure and function of cell membranes and plasma membranes, ultimately resulting in cell death [27].

Hogan *et al*. demonstrated significant expression of PUFAs in the serum of CCI mice, resulting in lipid peroxidation and ferroptosis [33]. Kenny *et al*. confirmed that the oxidation of PEs, protein expression alterations and glutathione (GSH) levels are consistent with the activation of ferroptosis following TBI [34]. In addition, a significant reduction in ferroptosis after TBI was observed by inhibiting 15-lipoxygenase, both *in vitro* and *in vivo*, indicating the potential to reduce histological and cognitive damage post-TBI [34]. Li *et al*. reported elevated cyclooxygenase-2 (COX-2) expression in a mouse model of intracerebral hemorrhage (ICH) [35], and the administration of the antioxidant Fer-1 via *in situ* or intraventricular injection inhibited the generation of lipid peroxides and COX-2 in the perihematomal tissue of ICH, thereby minimizing secondary brain injury [35]. These findings offer potential clinical treatment prospects for patients with ICH.

### The regulatory axis of the cystine/glutamate antiporter system Xc^−^/GSH/glutathione peroxidase 4

The cystine/glutamate antiporter system Xc^−^ (System Xc^−^) is a crucial intracellular antioxidant system [36]. It comprises solute carrier family 7 member 11 (SLC7A11) and solute carrier family 3 member 2 (SLC3A2) proteins, which form an amino acid reverse transport system embedded in the cell membrane [37,38]. SLC7A11 functions as the primary subunit, displaying high specificity for cystine and glutamate [38]. System Xc^−^ facilitates the 1 : 1 exchange of cystine into the cell and the efflux of glutamate [36]. Intracellular cystine is converted into cysteine through the catalytic activity of γ-glutamylcysteine synthetase, followed by the synthesis of GSH mediated by glutathione synthetase [16]. Inhibition of System Xc^−^ reduces intracellular cystine, resulting in decreased cysteine levels and disrupted GSH synthesis [36]. The tumor suppressor p53 downregulates SLC7A11 expression to inhibit cellular cystine uptake, thereby reducing glutathione peroxidase 4 (GPX4) activity, compromising the cell’s antioxidant capacity and increasing sensitivity to ferroptosis [39].

The upstream pathways of ferroptosis ultimately impact the activity of GPXs, directly or indirectly, resulting in a decline in cellular antioxidant capacity. This, in turn, leads to increased lipid peroxidation and elevated levels of lipid ROS and ultimately triggers ferroptosis [8,17]. Consequently, the GPX family plays a crucial role in the process of ferroptosis, with GPX4 assuming a more prominent role [16]. With the assistance of GSH, GPX4 can convert toxic lipid hydroperoxides (LOOH) into nontoxic lipid alcohols (LOH), effectively preventing the generation of lipid alkoxyl radicals (LO) and suppressing lipid ROS production [27]. This mechanism safeguards cells against the detrimental effects and potential mortality resulting from the accumulation of lipid peroxidation following TBI. Inactivation or silencing of GPX4 or its associated genes leads to the accumulation of lipid ROS and ultimately triggers ferroptosis [36]. Notably, (1S,3R)-RSL3 (RSL3), a significant inducer of ferroptosis, directly inhibits the activity of GPX4, leading to a reduction in cellular antioxidant capacity, an elevation in lipid ROS and ultimately triggering ferroptosis [40].

In a TBI mouse model, GPX4 displayed a significant decrease during the acute phase of TBI, reaching its lowest point at 12 h postinjury and returning to normal levels after 7 days [41]. Furthermore, a decrease in serum GSH was observed in clinically mild TBI patients, which correlated with the occurrence of posttraumatic epilepsy [42]. These studies suggest that the System Xc^−^/GSH/GPX4 pathway plays an important role in the activation of ferroptosis following TBI.

### Other regulatory pathways

The mevalonate pathway (MVA pathway) can act on GPX4 by regulating the maturation of selenocysteine tRNA, which ultimately triggers ferroptosis [43]. Selenocysteine, an essential amino acid in the active center of GPX4, necessitates the involvement of a specific transporter known as selenocysteine tRNA to integrate into GPX4 [44]. The maturation of selenocysteine tRNA involves the isopentenyltransferase that transfers the isopentenyl group from isopentenylpyrophosphate, a product of the MVA pathway, to the precursor of selenocysteine tRNA [45]. Therefore, the MVA pathway can downregulate isopentenylpyrophosphate, thereby affecting the synthesis of selenocysteine tRNA and further interfering with the activity of GPX4, leading to ferroptosis [43]. Statins that block the MVA pathway can interfere with the effective translation of GPX4, thereby increasing the sensitivity of cells to ferroptosis [46]. In addition, studies in ICH have found that simple intraventricular selenium administration can enhance the expression of GPX4, inhibit GPX4-dependent ferroptosis and significantly improve behavioral deficits in ICH rats [47]. Professor Liu Wanli and his collaborators revealed that intermediate metabolites of the MVA pathway can act as dangerous signals, triggering acute cell necrosis mediated by cytoplasmic calcium overload after extracellular calcium influx [48]. They have also preliminarily tested the clinical value of this metabolic pathway in brain infarction disease models. In recent years, the research team has published work revealing that the MVA pathway can serve as a drug target for vaccine adjuvant development [49].

The NAD(P)H/ferroptosis suppressor protein 1 (FSP1)/coenzyme Q10 (CoQ10) pathway is an alternative antioxidant pathway parallel to GPX4 [50]. CoQ10, a liposoluble antioxidant, can inhibit protein, lipid and DNA oxidation damage [51]. Within the mitochondrial membrane, FSP1, functioning as a redox enzyme, reduces ubiquinone (CoQ) to ubiquinol (CoQH2) [52]. CoQH2 acts as an antioxidant that scavenges lipid peroxides, thereby preventing their accumulation [53]. Consequently, inhibiting the expression of the NAD(P)H/FSP1/CoQ10 pathway can induce ferroptosis [50,54]. Currently, FSP1 is the sole known ferroptosis suppressor protein capable of compensating for GPX4 defects [52]. GPX4 has been documented to exhibit compensatory inhibitory effects on ferroptosis in FSP1 gene knockout mice [52,54]. Therefore, targeting FSP1 for TBI treatment holds great potential [55].

Nuclear factor-erythroid 2-related factor 2 (Nrf2) is a crucial factor in the antioxidant defense system [56] and is involved in timely protective responses against oxidative stress, inflammation and metabolic stress [57]. Upon exposure to oxidative pressure or specific chemicals, Nrf2 disengages from its binding partner, Kelch-like ECH-associated protein 1 (Keap1) [58,59], and binds to antioxidant response elements (AREs) located on the promoters of downstream target genes [56]. This action promotes the transcriptional activation of numerous antioxidant target genes, enabling the organism to exert antioxidant function and maintain redox homeostasis [57]. Nrf2 regulates hundreds of genes, many of which are directly or indirectly involved in the regulation of ferroptosis [56,57], such as superoxide dismutase 1 (SOD1), heme oxygenase 1 (HO-1), quinone oxidoreductase 1, GPXs, glutathione S-transferase and glutathione reductase [56]. These factors are crucial for Nrf2’s regulatory capacity in ferroptosis. In addition, Nrf2 has been proven to be a key neuroprotective factor against TBI in mice [60], and Nrf2 deficiency exacerbates neurological dysfunction and neuronal damage in mice after TBI [61]. Interestingly, increased nuclear translocation of Nrf2 and upregulation of HO-1 protein expression have been reported to significantly ameliorate TBI-induced behavioral deficits in TBI rats [62].

In addition to Nrf2, the mammalian target of rapamycin (mTOR) and the transsulfuration pathway are two other major mechanisms of resistance to ferroptosis. mTOR is a serine/threonine kinase with two complexes, mTORC1 and mTORC2 [63]. The fundamental role of mTOR revolves around the regulation of vital cellular processes encompassing proliferation, differentiation and migration. This regulation transpires via the canonical Phosphatidylinositol3-kinase (PI3K)/Serine/threonine protein kinaseB (AKT)/mTOR pathway, steering cells toward survival [64]. Notably, mTOR tends to become hyperactivated across diverse malignancies, a phenomenon linked to tumor progression [65]. Intriguingly, suppressing this pathway augments the efficacy of ferroptosis-mediated cancer therapy. Recent studies have found that mTOR is involved in the regulation of ferroptosis and can inhibit ferroptosis by downregulating lipid synthesis mediated by the SREBP1/SCD1 signaling pathway and affecting the generation of ROS in cells [66]. Therefore, targeting the mTOR signaling pathway may be an effective strategy for the treatment of related diseases. Zhang *et al*. reported that the inhibition of mTORC1 reduces the protein level of GPX4, sensitizes cancer cells to ferroptosis and cooperates with ferroptosis inducers to inhibit the growth of xenograft tumors derived from lung cancer patients in mice [67]. TBI damages the integrity of the BBB and releases albumin, which promotes the production of IL1β by glial cells and activates the mTOR pathway [68,69]. In animal models of TBI-associated epilepsy, the focal mTOR pathway is activated, and the application of rapamycin can reduce seizures and improve TBI-induced damage [70].

Certain mammalian cells can also utilize methionine as a sulfur donor in addition to extracellular uptake of cysteine and use the intermediates homocysteine and cystathionine to synthesize nascent cysteine through the transsulfuration pathway [71]. Thus, bypassing SystemXc^−^, such cells are resistant to ferroptosis induced by SystemXc^−^ inhibition [72]. Predominantly, research regarding the transsulfuration pathway has centered on cysteinyl-tRNA synthetase. Silencing cysteinyl-tRNA synthetase triggers the activation of the transsulfuration pathway, thereby enhancing cysteine synthesis within cells [73]. This augmentation effectively thwarts erastin-induced ferroptosis while leaving iron homeostasis unaffected [73]. Evidently, the transsulfuration pathway has a substantial influence on ferroptosis onset. Nonetheless, research concerning its involvement in TBI models remains sparse. The pivotal role of this pathway in TBI and the circumstances governing its activation warrant further investigation. Tchantchou *et al*. conducted a study to test the hypothesis that low-pressure-induced oxidative stress, coupled with alterations in homocysteine levels, contributes to the pathological progression of low-pressure-induced TBI [74].

### The roles of organelles in ferroptosis

Ferroptosis is a meticulously orchestrated process necessitating intricate signaling interactions among diverse organelles, encompassing mitochondria, the endoplasmic reticulum (ER), the Golgi apparatus and lysosomes, all of which are collaboratively engaged in regulatory functions [75].

Mitochondria, pivotal cellular components, play a multifaceted role in governing energy metabolism, signal transduction and apoptotic pathways [76]. Notably, they serve as the foremost generators of ROS and constitute the primary hub for intracellular iron-ion activity, thereby establishing an intimate interplay between ferroptosis and mitochondrial structure–function interrelationships [77]. A mounting body of empirical evidence underscores mitochondria’s pivotal role in driving ferroptosis by capitalizing on milieu-dependent metabolic influences [77]. Intriguingly, a deficit in cysteine induces hyperpolarization of mitochondrial membrane potential, culminating in the accrual of lipid peroxides [78], thereby setting off ferroptosis. Conversely, inhibition of the mitochondrial tricarboxylic acid cycle or electron transport chain can alleviate the repercussions stemming from cysteine deficiency [78]. Moreover, mitochondrial fatty acid metabolism furnishes the requisite lipid precursors indispensable for ferroptosis. This intricate process hinges primarily on bolstering proton conductance across the inner mitochondrial membrane, curtailing the electron transport chain, and triggering the opening of the mitochondrial permeability transition pore (mPTP), a regulatory entity orchestrating energy metabolism through these intricate mechanisms [79].

The ER serves as the central hub for protein synthesis, processing and lipid secretion [80]. ER stress prompts an unfolded protein response aimed at reinstating protein homeostasis; however, in instances where cellular equilibrium is not regained, it can culminate in cell death [81]. ER stress assumes a dual role in ferroptosis, contingent upon the diversity of ATF4 target genes. On the one hand, compounds such as erastin distinctly impede cystine uptake through System Xc^−^, inciting ER stress and consequentially instigating ferroptosis in a variety of cellular contexts: e.g. ATF4 participates in the upregulation of heat shock protein 5 and SLC7A11, thereby preventing the degradation of GPX4 and inhibiting ferroptosis [82]. On another note, in breast cancer cells, ATF4-mediated upregulation of γ-glutamylcyclotransferase 1 intensifies susceptibility to ferroptosis upon exposure to artemisinin or cystine deprivation [83]. Findings from research indicate that ER stress-associated Ca^2+^ influx, triggered by complex III accumulation at the membrane, averts membrane impairment during ferroptosis [83]. Importantly, Fer-1 potentially exerts its anti-ferroptosis effect through accumulation within the ER, as opposed to lysosomes and mitochondria.

Ferroptosis plays a pivotal role in regulating cell death triggered by Golgi stress, and ferroptosis inhibitors (AMF-26/M-COPA, brefeldin A and GCA) effectively suppress cell death, safeguarding cells from Golgi-related impacts while also influencing Golgi morphology and its mediated protein secretion [84]. Ferroptosis inducers (erastin, sorafenib, RSL3 and sulfasalazine) can mitigate lipid peroxidation induced by brefeldin A through the transsulfuration pathway, aiding in the restoration of Golgi homeostasis [84].

Lysosomes contain hydrolytic enzymes, such as the cathepsin family, capable of degrading and recycling essential nutrients for the organism [85]. Research findings indicate that lysosomes are involved in ferroptosis through three mechanisms: (1) activation of autophagy, (2) release of lysosomal cathepsins and (3) accumulation of lysosomal iron or NO [75]. Autophagy is a lysosome-dependent degradation pathway characterized by the formation of autophagosomes executed by autophagy-related genes (ATGs) [86]. Studies have shown that silencing ATGs can inhibit ferroptosis in many cancer cells [87]. Currently, the release of lysosomal cathepsins, particularly cathepsin B (CTSB), is considered one of the factors contributing to ferroptosis [88]. CTSB translocates from lysosomes to the cell nucleus, leading to DNA damage and subsequent STING1-dependent ferroptosis [89]. CTSB can also function as a specific histone H3 protease, cleaving H3 to induce ferroptosis [88]. Furthermore, the accumulation of lysosomal iron or NO can induce lipid peroxidation when reaching a certain concentration, consequently triggering lysosome-dependent ferroptosis [75].

### The role of ferroptosis in the chronic stage of TBI

**Figure 4:**
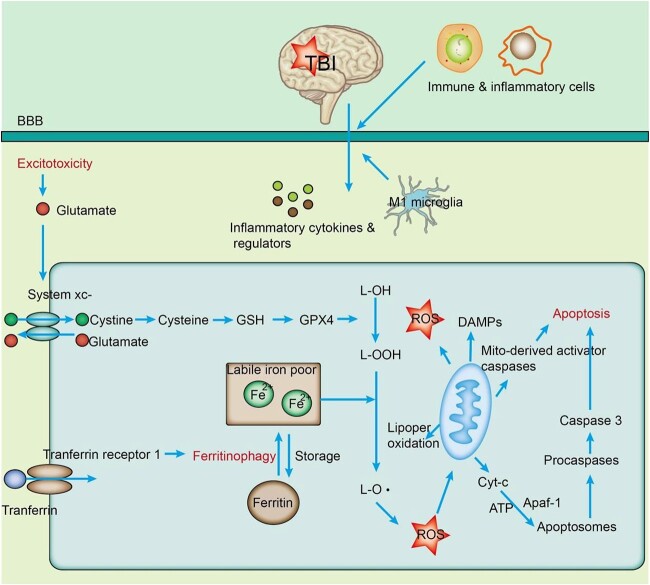
Secondary injury mechanisms and their interrelationships after TBI. After TBI, intracranial bleeding leads to iron deposition in the brain, disrupting iron metabolism. Activated microglia release toxic substances such as proinflammatory cytokines, complement proteins, proteases and other regulators, causing brain damage. Simultaneously, the BBB is disrupted, allowing immune and inflammatory cells such as neutrophils and macrophages to further infiltrate brain tissue, resulting in ROS production. Additionally, increased glutamate levels due to excitotoxicity impair the function of System Xc^−^, leading to the generation of more ROS, mitochondrial dysfunction, excessive free radical production, activation of caspase signaling pathways and promotion of cell apoptosis. Mitochondrial fragmentation, iron deposition and accumulation of lipid-derived ROS are characteristic features of ferroptosis. *TBI* traumatic brain injury, *BBB* blood–brain barrier, ROS reactive oxygen species, *GSH* glutathione, *GPX4* glutathione peroxidase 4

TBI is a chronic health condition that carries significant long-term consequences, including cognitive deficits, the development of neurodegenerative diseases, posttraumatic epilepsy and psychiatric disorders [90]. TBI yields profound and enduring effects on neurological function and mental well-being [91]. Multiple regulatory mechanisms prevail during secondary injury after TBI **(**Figure 4**).** After TBI, intracranial bleeding leads to iron deposition in the brain, disrupting iron metabolism. Activated microglia release toxic substances such as proinflammatory cytokines, complement proteins, proteases and other regulators, causing brain damage [92]. At the same time, the BBB is disrupted, allowing immune and inflammatory cells such as neutrophils and macrophages to further infiltrate brain tissue, resulting in ROS production [93]. Additionally, increased glutamate levels due to excitotoxicity can reportedly impair the function of System Xc^−^, leading to the generation of more ROS, mitochondrial dysfunction, excessive free radical production, activation of caspase signaling pathways and promotion of cell apoptosis [94]. Mitochondrial fragmentation, iron deposition and accumulation of lipid-derived ROS have been observed as characteristic features of ferroptosis. Thus, ferroptosis has been shown to play a significant role during the chronic stage of TBI [34].

### Traumatic brain edema

Traumatic brain edema is a secondary pathological process characterized by increased intracellular or extracellular fluid within the brain tissue due to external forces [95,96]. Patients with TBI often die from brain edema, suggesting that the therapeutic effect of brain edema directly determines the prognosis of this patient population [90]. The development of traumatic brain edema involves a combination of multiple factors, including inadequate cerebral perfusion, elevated intracranial venous pressure, disruption of the BBB and other factors that disturb the water balance within the brain tissue, leading to impaired water transport [96,97]. Consequently, the distribution of water and electrolytes across the brain cell membrane is disrupted, resulting in significant fluid accumulation inside and outside the brain cells [97,98]. Ultimately, this leads to increased brain volume, elevated intracranial pressure, and, in severe cases, the possibility of brain herniation or displacement [99].

The types of traumatic brain edema can vary and may present as a combination of different edema types that evolve over time following brain injury. These types often coexist with vasogenic and cytotoxic brain edema [95]. Vasogenic edema refers to the leakage of fluid and proteins into the brain tissue interstitium due to increased capillary permeability after disruption of the BBB, resulting in peritumoral brain tissue swelling and widened extracellular spaces [100]. Cytotoxic brain edema is a pathological condition characterized by cellular swelling and reduced extracellular spaces due to the inactivation of sodium–potassium ATPase in the cell membrane, leading to intracellular water and sodium retention [101].

Aquaporins (AQPs), also known as water channel proteins, are highly selective water channels widely distributed on the cell membranes of animals and plants [102]. The specific transmembrane protein family AQP4 is thought to play a crucial role in maintaining brain water balance and serves as a vital structural basis for water regulation and transport by astrocytes, cerebrospinal fluid and blood vessels [103,104]. Current research suggests a close relationship between AQP4 and the formation of traumatic brain edema [104]. The expression and distribution of AQP4 undergo corresponding changes as the duration of TBI increases and brain edema progresses [105]. Additionally, TBI can induce the upregulation of Nuclear factor kappa-B (NF-κB), IL-1β, IL-6, tumor necrosis factor-α (TNF-α) [106] and other target genes involved in ferroptosis, as well as the expression of AQP4 [104], leading to increased disruption of the BBB and exacerbation of traumatic brain edema. The ferroptosis inhibitor Fer-1 has been shown to reduce the levels of IL-1β and TNF-α and BBB disruption, thereby mitigating brain edema [55].

Following TBI, the excessive generation of ROS disrupts the BBB, triggering brain edema [96]. Under ischemic and hypoxic conditions, brain cell damage leads to the release and accumulation of glutamate in the extracellular space, causing a rapid elevation in intracerebral glutamate concentration [107]. This increases neuronal membrane permeability to ions, allowing a large influx of Ca^2+^ into the cells, resulting in intracellular Ca^2+^ overload [108]. Simultaneously, this process activates Na^+^ channels, promoting an increased influx of Na^+^ and elevated intracellular osmotic pressure, ultimately culminating in cytotoxic brain edema [109]. In addition, the excessive expression of GPX4 has been shown to mitigate brain edema and prevent BBB disruption in TBI animal models [110]. The elevation of arachidonic acid and PUFAs in the brain contributes to increased water and sodium levels [111], while the reduction in potassium and ATP-dependent Na^+^/K^+^ pumps contributes to the development of brain edema [109].

### Cognitive dysfunction

TBI is one of the important causes of cognitive dysfunction [112]. It has been reported that patients experience a marked decline in cognitive abilities, including immediate recall, short-term memory, long-term memory, working memory and attention, within 3–5 days after sustaining a brain injury [113,114]. Some cognitive impairments can even be detected within the first year following TBI [113,115]. Additionally, as individuals age, subtle structural and functional changes occur in the brain, rendering the brains of older individuals more vulnerable to the effects of TBI. These age-related changes decrease plasticity and reparative potential following cranial injuries in older adults, resulting in more persistent cognitive impairments [116]. The pathophysiological molecular mechanisms underlying cognitive dysfunction caused by TBI are complex and primarily involve central nervous system inflammation, oxidative stress, free radical damage and disrupted metabolism of neuroprotective factors [117].

TBI-related cognitive dysfunction has been associated with dementia and neuropathological features commonly observed in AD [118], such as amyloid plaque deposition and tau neurofibrillary degeneration [119,120]. Abnormalities in iron metabolism are prevalent in TBI patients with cognitive impairments [24], and increased brain iron content is correlated with cognitive decline; therefore, ferroptosis is widely thought to play a significant role in cognitive dysfunction following brain trauma [24]. A recent study by Rui *et al*. demonstrated that melatonin could mitigate TBI-induced brain edema and cognitive impairments by inhibiting inflammation and ferroptosis, providing a new avenue for rehabilitation treatment and prognosis assessment in TBI patients [121]. In a CCI mouse model, intraventricular injection of Fer-1 was found to significantly reduce neuronal degeneration and improve long-term cognitive function [122].

It is well established that the clinical hallmark of AD is cognitive impairment. A study by Plassman *et al*. on World War II veterans found that moderate to severe TBI was associated with a significantly increased incidence of AD and dementia [123]. A population-based study demonstrated that individuals who experienced TBI before age 65 experienced a significantly earlier onset of AD than those without a history of TBI [124]. Following TBI, similar characteristics to ferroptosis are present in AD, including iron overload, lipid peroxidation, GPX4 inactivation and BBB damage [125]. These findings provide evidence for the involvement of ferroptosis in the development of cognitive impairments after trauma, although further investigation is required to elucidate the specific mechanisms.

### Psychiatric disorders

In clinical practice, psychiatric disorders caused by TBI are relatively common, primarily involving frontal lobe contusions and cerebral hemorrhage [126]. The frontal lobe, known as the emotional center of the human body [127], is susceptible to damage, which can lead to emotional control disorders such as schizophrenia, mania, aggression and verbal abuse, ultimately manifesting as psychiatric conditions [128]. Depression is a severe mental illness that poses a significant threat to human health [129], and its pathogenesis is complex and diverse, with oxidative stress and inflammation being important pathological mechanisms [130,131]. Extensive evidence supports a close association between oxidative stress and depression.

In cases of depression following TBI, a decrease in antioxidants and an increase in ROS have been observed, contributing to an imbalance in the body’s oxidative and antioxidative systems [132,133]. In patients with depression or animal models, significant alterations in iron content or iron-related genes associated with ferroptosis have been observed [134], indicating that abnormal iron metabolism in the brain may contribute to the pathophysiological changes of depression [11,134]. Moreover, clinical and animal experimental data suggest that antidepressant drugs that regulate ferroptosis can eliminate ROS and reactive nitrogen species by clearing free radicals and inhibiting the oxidative stress pathway, thereby protecting neurons from oxidative stress damage and treating depression [135,136]. For example, edaravone (EDA) can improve the mood of depressed mice by increasing the expression of silent information regulator 1 (SIRT1), Nrf2, HO-1 and GPX4 in the hippocampus of mice [137]. Similarly, ferroptosis features have been documented in Huntington’s disease [138], and intraventricular injection of the iron chelator deferoxamine (DFO) was found to improve the pathological features and motor phenotype of R6/2 HD mice [139]. The above studies overlap in their assertion that ferroptosis represents a potential therapeutic target for psychiatric disorders.

### Posttraumatic epilepsy

Posttraumatic epilepsy (PTE) refers to the occurrence of focal or generalized seizures as a result of trauma that can be categorized as early-onset epilepsy (within 1 week after injury) or late-onset epilepsy (1 week to several years after injury) [140,141]. The primary characteristic of epilepsy is abnormal neuronal discharge, which can induce various pathological changes in cells [141]. Excessive activation of oxidative stress is a typical pathological feature of epilepsy [142]. It is well recognized that injecting hemoglobin or iron salts into the cortex can establish an animal model of chronic epileptic foci. Mori *et al*. documented increased intracellular superoxide anions and hydroxyl radicals following intracortical injection of ferric chloride [143]. Subsequently, activation of neuronal membrane lipid peroxidation leads to recurrent seizures [143]. These findings suggest that iron overload is a prevalent factor contributing to the development of posthemorrhagic epilepsy and PTE.

Furthermore, treatment with the iron chelator DFO in a ferric chloride-induced epilepsy animal model has been shown to reduce local transferrin levels and significantly suppress seizure activity [144]. Mueller *et al*. found lower levels of GPX4 and GSH in epilepsy patients than in healthy controls [145]. Additionally, Shekh-Ahmad *et al*. demonstrated that inhibiting Keap1 and activating Nrf2 with Omaveloxolone (RTA408) can prevent the accumulation of ROS, mitochondrial depolarization and neuronal death during seizure activity, suggesting that targeting the antioxidant system may serve as a therapeutic approach for seizure prevention [146]. In conclusion, the regulation of iron, lipid peroxidation and GSH/GPX4 levels significantly influences ferroptosis in patients with PTE [147].

### Therapeutic interventions targeting ferroptosis following TBI

Iron deposition, lipid peroxidation and GSH depletion in ferroptosis are closely related to the long-term consequences of neurodegeneration and neurological impairment following TBI. Next, we comprehensively review advancements in TBI treatment, emphasizing targeting ferroptosis and providing valuable insights for developing TBI therapeutics (Table 1).

**Table 1:** Ferroptosis-related drugs used to treat TBI

**Drugs**	**Classification**	**Neuroprotective effects**	**Potential mechanism**	**References**
Deferoxamine	Iron chelator	Reduces the volume of brain injury and improves cognitive impairment	Reduces iron deposition	[166,167]
Deferiprone	Iron chelator	Enhances learning and memory	Reduces iron level	[169,170]
*N*,*N*′-bis(2-Hydroxybenzyl)ethylenediamine-*N*,*N*′-diacetic acid	Iron chelator	Improves motor function and exerts neuroprotective effects	Reduces iron level and multifunctional antioxidant activity	[173]
Minocycline	Iron chelator	Alleviates neural damage	Reduces iron deposition	[176,178]
Ferrostatin-1	Lipid peroxidation inhibitor	Attenuates neurodegeneration; ameliorates long-term motor and cognitive prognosis	Reduces iron deposition	[23]
Baicalein	12/15-LOX inhibitor	Antioxidant, anti-inflammatory and improves learning and memory	Suppresses the 15-LOX pathway	[197]
Liproxstatin-1	Lipid peroxidation inhibitor	Attenuates the degenerating neurons and motor performance; improves cognitive dysfunction	Increases GSH, inhibits System Xc^−^, decreases iron and lipid oxidation	[186,187]
Melatonin	Hormone	Improves the neuron's survival and motor performance	Upregulates GSH, reduces iron and lipid oxidation	[121]
miR-212-5p agonist	PTGS2 inhibitor	Improves learning and spatial memory	Suppresses the enzyme catalyzing lipid oxidation	[10]

*TBI* traumatic brain injury, *GSH* glutathione, *PTGS2* prostaglandin-endoperoxide synthase 2, *15-LOX* 15-lipoxygenase

### Activation of the Nrf2 pathway

In recent years, targeted Nrf2 therapy for TBI has emerged as a prominent research hotspot due to its potential in alleviating oxidative stress and inhibiting ferroptosis [41,60,61]. Currently, the main therapeutic drugs under investigation include dexmedetomidine, ketamine, EDA, curcumin, fucoxanthin and tert-butylhydroquinone (TBHQ) [148]. Li *et al*. demonstrated that dexmedetomidine could elevate the expression levels of Nrf2, HO-1 and quinone oxidoreductase 1 in TBI patients, thereby suppressing the production of inflammatory factors and reducing cell death caused by neuroinflammation [149]. In addition, growing evidence suggests that ketamine can alleviate post-TBI oxidative stress and exert neuroprotective effects through Nrf2 activation [150,151]. In addition, the combined administration of ketamine with other sedatives improved analgesic and sedative effects in TBI patients, exhibiting good safety profiles [152]. Treatment with EDA in TBI rat models reduced the injury area, alleviated hippocampal damage, enhanced memory and learning abilities, and activated Nrf2 [153]. Curcumin, a natural phenolic compound, possesses various pharmacological effects, including anti-inflammatory and antioxidant properties [154]. Dong *et al*. found that curcumin can mitigate cortical injury in TBI mice, inhibit neutrophil infiltration, suppress microglial cell activation, and attenuate neuronal apoptosis and degeneration following TBI. However, the neuroprotective effect of curcumin is attenuated in Nrf2 gene knockout mice following TBI [155]. The presence of fucoxanthin in seaweed has been established to confer potent antioxidant activity [156]. In this respect, Zhang *et al*. discovered that fucoxanthin could activate Nrf2 both *in vitro* and *in vivo* [157]. TBHQ serves as an Nrf2 activator, and studies have demonstrated that intervention with a combination of vanillin acetate and TBHQ in a TBI mouse model can activate Nrf2-related pathways, alleviate oxidative stress and protect the gray matter of the mouse brain [158,159]. However, despite the potential of these drugs to alleviate TBI through Nrf2 activation, most studies have been conducted on animal models, necessitating extensive clinical trials to further evaluate their efficacy.

### Iron chelation therapy

Given that iron overload has been documented in the brain tissue of TBI patients, removing excess deposited iron through medication may be an effective treatment method to improve the prognosis of TBI patients [160,161]. Recent studies have highlighted the significant role of iron chelators in providing brain protection for TBI patients. Medical iron chelators can be classified into three categories: iron carriers, synthetic chelators and natural chelators [161,162].

DFO can bind to excessive accumulated iron in tissues and cells under pathological conditions [163]. It undergoes oxidative deamination and is excreted through the kidneys in the urine [164]. DFO is extensively utilized to treat iron overload disorders [163]. Clinical trials have shown that DFO can promote the absorption of hematoma and edema following TBI and mitigate neuronal degeneration, myelin damage and inflammation after experimental cerebral hemorrhage [165]. In TBI mouse models, DFO can alleviate iron-induced long-term neurotoxicity and acute brain edema resulting from TBI [166,167]. Additionally, the combined administration of dextran and DFO in a severe TBI mouse model improved the grip strength and prognosis of the mice [168]. Consequently, DFO holds significant application value in the treatment of TBI.

Deferiprone is an oral iron chelator commonly used in clinical settings to reduce iron levels in patients suffering from chronic conditions such as transfusional iron overload [169]. It exerts iron-chelating effects by directly binding to serum iron ions [170]. In recent years, deferiprone has shown enormous prospects in reversing brain iron toxicity and improving treatment outcomes [171]. The combination of deferiprone with acetylcysteine could significantly enhance learning and memory abilities in iron-overloaded rats and promote mitochondrial function and preservation of BBB integrity [172]. Nevertheless, additional research is required to validate its therapeutic efficacy in TBI.

*N*,*N*′-bis(2-Hydroxybenzyl)ethylenediamine-*N*, *N*′-diacetic acid (HBED) functions as a hydrogen donor and inhibitor of hydroxyl radicals [173]. It can directly traverse the BBB and mitochondrial membrane, allowing it to bind with ferrous ions, converting them into ferric ions, and alleviating brain damage caused by ferrous ions [174]. In a TBI mouse model, treatment with HBED reduced microglial proliferation and AQP4 expression, thereby alleviating secondary damage in the cortical region of mice following TBI [175]. Consequently, it improves motor function in mice and exhibits neuroprotective effects [175].

Minocycline is a semisynthetic tetracycline antibiotic with high lipid solubility, facilitating penetration of the BBB [176]. Multiple studies have confirmed that minocycline can exert neuroprotective effects in diseases such as cerebral hemorrhage, cerebral ischemia and TBI [177]. Following TBI, minocycline can diminish iron concentrations in the cerebrospinal fluid, cortical tissue and hippocampus [178]. It also inhibits the excessive expression of iron proteins and TFR1 in the hippocampus and cortex, thereby regulating iron metabolism [178]. Therefore, it is evident that minocycline plays an important role in alleviating the neural damage caused by TBI.

Given the nephrotoxicity and cardiotoxicity associated with iron chelators, their usage is currently limited to experimental settings [179]. Furthermore, there is a need for additional research to explore the clinical applicability and long-term outcomes of iron chelation therapy in TBI patients [180].

### Other therapies

Fer-1 has previously been identified as an effective and specific inhibitor of ferroptosis [181,182]. Moreover, studies have indicated that Fer-1 inhibits cell death in various disease models, including HD, acute brain injury, periventricular leukomalacia and renal failure, and improves stroke and PD models by inhibiting glutamate-induced ferroptosis [183,184]. In addition, intraventricular injection of Fer-1 in TBI models has been shown to reduce iron accumulation, alleviate neuronal degeneration, and improve cognitive and motor impairments caused by TBI [185]. However, other clinically applicable administration methods, such as intraperitoneal or intravenous administration, warrant validation in further studies.

Liproxstatin-1 (Lip-1) is a potent antioxidant that effectively scavenges free radicals and restores the expression of GPX4 and GSH [186,187]. It inhibits mitochondrial lipid peroxidation and rescues cells from ferroptosis [186,187]. Lip-1 has demonstrated potent inhibition of ferroptosis at low nanomolar concentrations, exhibiting superior efficacy to ferroptosis inhibitors such as DFO and Fer-1 [186]. In recent studies, statin drugs have been found to significantly mitigate functional deficits in neural tissue following TBI [188,189]. Additionally, Feng *et al*. demonstrated that Lip-1 could effectively inhibit GPX4-induced ferroptosis in oligodendrocytes, highlighting its potential therapeutic value in the repair of the central nervous system [187].

Baicalein (BAI) is a natural flavonoid compound in Scutellaria baicalensis, a plant species [190]. Compared to synthetic antiferroptotic agents such as Lip-1 and Fer-1, BAI offers the advantage of facilitating clinical implementation [191]. Research has revealed that BAI exhibits diverse physiological and pharmacological effects, including antioxidant, anticancer, anti-inflammatory, antibacterial, antidepressant, antiviral and sedative activities [192,193]. BAI can be used to treat cerebral ischemia [194] and it reduces the cerebral infarct area and improves neurofunctional deficits in focal cerebral ischemia rats [194]. Additionally, BAI significantly enhances motor function scores and improves motor coordination in animal models after cerebral ischemia [195], potentially through its involvement in regulating synaptic plasticity and axonal growth [196]. Moreover, BAI is a 12/15-lipoxygenase (12/15-LOX) inhibitor capable of reducing damage caused by ferroptosis and improving behavioral changes caused by TBI [197].

Melatonin is an amine hormone derived from tryptophan that is primarily synthesized by the pineal gland in mammals and humans [198]. Current research has revealed that melatonin possesses potent antioxidant properties, anti-inflammatory effects and the ability to penetrate the BBB [199,200]. Its lipophilic nature facilitates effortless diffusion across cell membranes and entry into subcellular compartments, thereby impeding a broad spectrum of reactions triggered by TBI, including the ferroptosis pathway [201]. Rui *et al*. found that melatonin effectively enhances cognitive and motor function post TBI by inhibiting ferroptosis mediated by the heavy chain of ferritin [121].

*miR-212-5p* is a type of Micro ribonucleic acid (miRNA) widely expressed in various tissues [202]. miRNAs play a crucial role in brain development and the establishment of complex neural networks [203]. Abnormalities in miRNAs have been associated with neurodegenerative diseases. In addition, different brain pathologies, including ischemic brain injury and TBI, can lead to changes in miRNA levels, affecting patient prognosis [204,205]. Xiao *et al*. discovered that overexpression of *miR-212-5p* attenuates ferroptosis, while downregulation of *miR-212-5p* promotes ferroptosis by targeting prostaglandin-endoperoxide synthase 2 (PTGS2) in HT-22 and Neuro-2a cell lines [10]. The use of *miR-212-5p* in mice with controlled cortical impact-induced TBI significantly improves learning and spatial memory [10], suggesting that miR-212-5p may prevent neuronal ferroptosis in controlled cortical impact mice by targeting PTGS2.

### Present challenges and future prospects

TBI is a serious global public health problem characterized by elevated mortality rates, complex treatment challenges, poor prognoses and long-term consequences encompassing brain edema, inflammation, neurodegeneration, motor impairments and cognitive deficits [90,141]. These outcomes can lead to severe disability and death, imposing a substantial economic burden on individuals and families [206]. However, the pathogenesis of TBI is highly complex, with many mechanisms yet to be explored, and there are currently no clinical guidelines or recommendations for its management [4,207,208].

Recent studies have demonstrated that secondary brain injuries resulting from TBI, including disruptions in ion channels, excitotoxicity, iron release, oxidative stress, lipid peroxidation, ROS accumulation and mitochondrial dysfunction, are closely linked to ferroptosis [6,55]. Ferroptosis is a novel form of programmed cell death characterized by toxic lipid peroxidation and increased intracellular levels of ROS, which depend on the accumulation of intracellular iron [8,17]. It differs from apoptosis, necrosis and autophagy and possesses unique regulatory mechanisms [8,17]. The sensitivity of ferroptosis is closely related to many biological processes, including amino acid, iron and polyunsaturated fatty acid metabolism, as well as the biosynthesis of GSH, phospholipids, NADPH and CoQ10. It is also associated with pathological cell death related to mammalian degenerative diseases (such as AD, HD and PD), tumors, stroke, cerebral hemorrhage, TBI, local ischemia–reperfusion injury and renal failure [209,210]. The discovery of ferroptosis has opened up a new platform for disease research, and its clinical significance in the occurrence, development and treatment of diseases is gradually emerging. At present, there is increasing research progress in understanding ferroptosis, but it is still in its infancy and many issues remain unresolved. The role of ferroptosis in human diseases is still unclear. What is the relationship between ferroptosis and other brain-specific cell death pathways (such as apoptosis and autophagy)? Do these different cell death modes form interactive signaling pathways? These issues still need further exploration. A deeper understanding of the relationships between these pathways will bring new hope for addressing refractory diseases. In addition, the susceptibility of cells in different tissues to ferroptosis varies greatly. Similarly, the sensitivity of cells to ferroptosis inducers varies among different individuals. Research on the role of organelles in ferroptosis is also not comprehensive. How do organelles communicate when ferroptosis occurs? How can molecular probes related to ferroptosis be developed to dynamically monitor changes in cellular morphology and function? Does the occurrence and transformation of specific organelles affect cellular susceptibility to ferroptosis? Therefore, elucidating the roles of organelles in ferroptosis constitutes an appealing research area that could provide new directions for disease treatment.

Our exploration of these processes in TBI is still in the early stages. Further research is needed on the specific regulatory mechanisms of ferroptosis on clinical manifestations in TBI, as well as the role of iron in ROS generation and neurotoxicity. Therefore, understanding the role of ferroptosis in TBI can provide potential therapeutic targets for its treatment [211]. Currently, research on the relationship between TBI and ferroptosis is limited, posing a significant obstacle to TBI research, drug development and subsequent clinical implementation [211]. Recent studies have confirmed the efficacy of iron chelators, ferroptosis inhibitors and Nrf2 activation in ameliorating secondary damage after TBI [34]. Nonetheless, existing studies still have several limitations, including short treatment durations, limited observation indicators to assess prognosis improvement, a small sample size of clinical cases and the need for further clarification of potential side effects [212]. Due to their toxicity to the kidneys and heart, iron chelating agents are only used in experiments. The clinical application and long-term prognosis of iron chelation therapy in patients with brain injury are also worth further research. Therefore, broader experimental research and subsequent clinical trial results are warranted to provide robust evidence before comprehensive clinical application. In addition, animal experiments investigating the relationship between ferroptosis and TBI typically use inhibitors such as ferritin to block cell death, but current research has not yet elucidated the specific signals that induce ferritin deposition in complex brain environments. Therefore, a fundamental area of future research involves biochemical regulation of iron conversion processes in brain cells.

## Conclusions

Exploring the pathogenesis of ferroptosis and its role in TBI and proposing effective therapeutic approaches can contribute to theoretical innovation in TBI clinical research and present new avenues and possibilities for TBI treatment.
